# In vivo performance of electrospun tubular hyaluronic acid/collagen nanofibrous scaffolds for vascular reconstruction in the rabbit model

**DOI:** 10.1186/s12951-021-01091-0

**Published:** 2021-10-30

**Authors:** Yuqing Niu, Massimiliano Galluzzi, Ming Fu, Jinhua Hu, Huimin Xia

**Affiliations:** 1 Department of Pediatric Surgery, Guangdong Provincial Key Laboratory of Research in Structural Birth Defect Disease, Guangzhou Women and Children’s Medical Center, Guangzhou Medical University, Guangzhou, 510623 Guangdong People’s Republic of China; 2 Materials Interfaces Center, Shenzhen Institutes of Advanced Technology, Chinese Academy of Sciences, Shenzhen, 518055 Guangdong People’s Republic of China

**Keywords:** Hyaluronic acid, Collagen, Blood vessel, Scaffold, Patency

## Abstract

One of the main challenges of tissue-engineered vascular prostheses is restenosis due to intimal hyperplasia. The aim of this study is to develop a material for scaffolds able to support cell growth while tolerating physiological conditions and maintaining the patency of carotid artery model. Tubular hyaluronic acid (HA)-functionalized collagen nanofibrous composite scaffolds were prepared by sequential electrospinning method. The tubular composite scaffold has well-controlled biophysical and biochemical signals, providing a good matrix for the adhesion and proliferation of vascular endothelial cells (ECs), but resisting to platelets adhesion when exposed to blood. Carotid artery replacement experiment from 6-week rabbits showed that the HA/collagen nanofibrous composite scaffold grafts with endothelialization on the luminal surface could maintain vascular patency. At retrieval, the composite scaffold maintained good structural integrity and had comparable mechanical strength as the native artery. This study indicating that electrospun scaffolds combined with cells may become an alternative to prosthetic grafts for vascular reconstruction.

**Supplementary Information:**

The online version contains supplementary material available at 10.1186/s12951-021-01091-0.

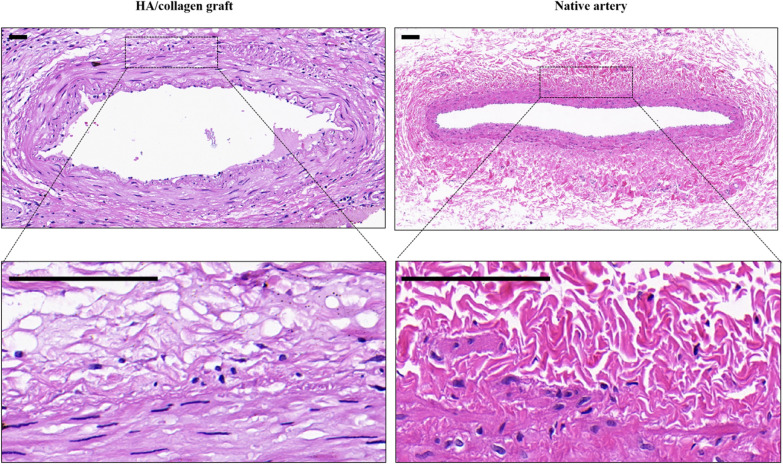

## Introduction

Cardiovascular diseases are the leading causes of death worldwide [1, 2]. Autogenous vein is still the gold standard of vascular bypass. However, in 30% of patients, there is no suitable vein, and the use of artificial vascular graft is considered to be a promising way to solve the limitations of current treatment methods [3]. Tissue-engineered blood vessels, constructed by cells and biomaterial scaffolds, are promising substitutes for native blood vessels [4, 5]. In this method, autologous vascular endothelial cells (ECs) are implanted on the luminal surface of biomaterial scaffolds, and a monolayer of ECs is formed before implantation [6]. Nevertheless, in small-diameter vascular graft applications, the proliferation of vascular ECs along the biomaterial scaffolds luminal surface is impaired, which leads to the failure of vascular graft patency in the early stage of implantation [7]. To promote the efficiency of endothelialization, a variety of biomaterials, such as extracellular matrix (ECM)—based proteins [8–10] and glycosaminoglycans (GAGs) [11], are widely used to prepare vascular scaffolds promoting the proliferation of vascular ECs [10, 12].

Native vessel is composed of anti-thrombogenic luminal surface lined by a monolayer EC and a thick external media composed primarily of vascular smooth muscle cells (SMCs) [13, 14]. From the perspective of tissue engineering, it is more and more evident that ECM molecules provide the necessary biomechanical and biochemical stimulation for cultured cells to create an environment mimicking the native tissues [15]. In the field of cell culture scaffolds, several studies evidenced that hyaluronic acid (HA) has great specificity in regulating the proliferation of ECs [16–18], a key point to the successful endothelialization of transplanted blood vessels [16, 19]. Therefore, it is feasible to immobilize HA with small molecular weight on the inner surface of biomaterial scaffold to promote endothelialization. Collagen is the main component of ECM in native blood vessels [14]. Because of its wide availability, good biomechanical properties and simple processing methods, it is widely used as a scaffold biomaterial for tubular organs [9, 10, 20, 21]. The customized collagen scaffold produced by electrospinning technology can achieve the similar topology architecture of ECM [20–22]. Moreover, electrospinning can produce scaffold materials with specific components, so as to achieve controlled degradation in the remodeling process [23, 24].

Tissue engineering in vascular reconstruction has shown considerable advancements [4, 9, 25]. However, it is still uncertain whether tissue-engineered vascular scaffolds can support cell growth and tolerate physiological conditions while maintaining vascular patency in vivo with active host tissue response. Regeneration is a cooperative process of cell growth, differentiation and histomorphogenesis [26]. To reveal this, tubular HA/collagen nanofibrous composite scaffolds were obtained by sequential electrospinning and cross-linking. The scaffold consisted of a thin inner layer and a thick outer layer, to mimic the intima and media of native blood vessels, respectively. Formation of a thin HA nanofibrous layer on the inner wall of the scaffolds help to optimize the surface biochemistry of scaffolds for vascular ECs’s endothelialization, while pure collagen nanofibers on the outer layer can provide mechanical support for cell growth. This allows us to hypothesize that tubular HA/collagen nanofibrous composite scaffolds can have beneficial effects on the growth, proliferation, spatial distribution, and phenotypic shape maintenance of vascular cells under physiologic conditions.

To verify the above hypothesis, we investigated whether the tubular HA/collagen nanofibrous composite scaffold can maintain vascular cells under physiological flow conditions in vivo.

## Experimental sections

### Materials and reagents

Hyaluronic acid (Mw ≈ 800 kDa, F1177), collagen (11179179001), plasminogen (341578), Dulbecco’s modified Eagle’s medium (DMEM, D0819), 4′6-diamidino-2′-phenylindole dihydrochloride (DAPI, 10236276001), phosphate buffered saline (PBS, pH 7.4, P4417), bovine serum albumin (BSA, A3858), thiazolyl blue (Synonyms: MTT, HY-15924), paraffin (327204), and dehydrated alcohol (1012772) were obtained from Sigma-Aldrich. Fetal bovine serum (FBS, 16140071), penicillin/streptomycin (P/S, 15070063), trypsin (0.25%, 15050065), CD31 monoclonal antibody (37-0700), live/dead assay kit (L3224) were obtained from Gibco. Anti-alpha (ɑ)-smooth muscle actin polyclonal antibody (α-SMA, ab5694) and cell counting kit-8 (CCK-8, ab228554) were purchased from Abcam. 4% paraformaldehyde (C104190), verhoeff-van gieson (VVG, GP1035), Triton X-100 (WGT8200), hematoxylin eosin (H&E, G1004, G1002) stain kit, secondary antibodies, Alexa Fluor-488, or Cy3-conjugated anti-mouse or anti-rabbit immunoglobulin-G (IgG) for fluorescence staining were obtained from Servicebio Science & Technology Co., Ltd. Hexafluoroisopropanol (HFIP, 920-66-1) was purchased from Aladdin.

### Preparation of HA/collagen nanofibrous scaffolds

Collagen (10%) and HA (2%) electrospinning solutions were prepared, respectively, by dissolving 1 g of collagen and 0.2 g of HA in 10 mL of HFIP. Firstly, 2% HA electrospinning solution was transferred into a 10 mL syringe and fixed on the precise injection pump of the electrospinning machine. The feeding rate of the solution was set at 0.36 mL/h, while the voltage power supply at 11 kV, and the distance between the spinneret tip and the grounded drum collector was fixed at 10 cm. A stainless-steel rod with diameter of 3 mm was used as collector maintaining a rotation rate at 1200 rpm. The electrospinning environment was maintained at 18–25 °C and 40–45% (relative humidity). The final structure of layered tubular nanofibrous scaffold was obtained by electrospinning HA solution for 20 h, followed by pure collagen spinning solution for 40 h employing the same electrospinning parameters. For comparison, tubular pure collagen nanofibrous scaffold with similar parameters was also prepared. The stability and strength of both tubular HA/collagen composite and pure collagen nanofibrous scaffolds were improved by crosslinking in 2.5% glutaraldehyde water vapor for 6 h, and then sterilized with ultraviolet lamp for 2 h before further uses.

### Characterizations

The morphology of electrospun tubular HA/collagen nanofibrous scaffolds was observed by scanning electron microscopy (SEM, SU8010, Hitachi) with an accelerating voltage of 5 kV after sputter coating with gold. Image analysis software ImageJ (http://imagej.net/citing) was used to measure the average pore size distribution of electrospun nanofibers from SEM images. For each sample, 120 pores were randomly selected from 5000$\times$ magnification images [27–29]. The porosity and diameter of the nanofibers of the tubular HA/collagen scaffolds were calculated using our previously published method [30].

The dynamic swelling of tubular HA/collagen and collagen nanofibrous scaffolds were analyzed by calculating the expansion rate of each sample using the following formula:

$$ {\text{Swelling}}\;{\text{rate}}\;\left( \% \right) = {{w_{{\text{s}}} w_{{\text{d}}} } \mathord{\left/ {\vphantom {{w_{{\text{s}}} w_{{\text{d}}} } {w_{{\text{d}}} }}} \right. \kern-\nulldelimiterspace} {w_{{\text{d}}} }} \times 100\% , $$

where *w*_s_ and *w*_d_ are the swollen weight and dry weight of each sample, respectively.

The tubular HA/collagen nanofibrous scaffolds before and after crosslinking were characterized by Fourier transform infrared spectroscopy (FTIR) with attenuated total reflection (ATR) head (ATR-FTIR, thermo Nicolet, Waltham, MA) using a scanning resolution of 2 cm^−1^ in the range of 500–4000 cm^−1^ [31, 32].

Tensile strength of the grafts was measured before implantation and 1 month after implantation, and the values were compared with the native rabbit carotid artery. Mechanical testing of these samples was performed by a tensile testing instrument (Sans, Shenzhen, China), equipped with a 50 N load cell [32–34]. Samples from pre-implant and post-implant grafts (*n* = 3) were cut into strips of 9.4 mm $\times$ 40 mm for testing. Samples were tested with a strain rate of 2 mm/min and peak stress at rupture was calculated and plotted.

### Cell culture

Primary vascular ECs were isolated from New Zealand rabbits using a CD133 affinity based blood purification system. The recovered cells were seeded in tissue culture 6-well plates and cultured in DMEM containing 10% FBS, 1% P/S until ECs colonies were formed. Primary smooth muscle cells (SMCs) were isolated from New Zealand rabbit carotid artery explants. The DMEM containing 15% FBS, and 1% P/S. SMCs are allowed to migrate from the chopped tissue. Then the ECs and SMCs were cultured and expanded in a conventional way.

Cells were cultured at 37 °C in an incubator with a humidified 5% CO_2_ atmosphere. The culture medium was changed every 2 days. The vascular ECs and SMCs were used at passage 3 for further experiments.

### Culture of vascular ECs and SMCs in tubular scaffolds

Tubular HA/collagen nanofibrous scaffolds (5 cm long, 3 mm inner diameter) were extensively washed in 1$\times$ PBS for 1 h, and then sutured at both ends with 5–0 polyglycaprone 25 (MONCRYLTM) suture to prevent cell extravasation. Next, vascular ECs with a density of 1$\hspace{0.17em}\times$ 10^6^ cells/mL were injected into the lumen of the scaffolds via a 25 G microlancetm (BD, Drogheda, Ireland) needle. The cell scaffold was transferred to a 50 mL tube containing 25 mL medium and rotated at 10 rpm in an incubator at 37 °C and 5% CO_2_ atmosphere. After 24 h, the sutures were removed, and the endothelialized scaffold was transferred to a new 50 mL tube containing 25 mL medium. SMCs with a density of 10^6^ cells/mL were seeded on the outer surface of the scaffolds and rotated at 10 rpm. The culture medium was changed every other day. In general, the electrospun vascular constructs were treated for 7 days before evaluation by histology and SEM.

### Platelet adhesion test

After exposing the carotid artery of New Zealand rabbits by operation, a cannula was inserted into the proximal and distal part of the artery to facilitate blood to enter and form blood circulation. Cell seeded and cell-free electrospun grafts were mounted on a Ruhr connector and placed between two cannulas. In this operation, arterial blood flowed through each sample for 15 min, and then the samples were gently rinsed three times with normal saline to remove non adherent blood platelets. After that, each sample was fixed with 4% formaldehyde for 15 min, and dehydrated with ethanol gradient solutions (85%, 95% and 100%, v/v). After freeze-drying, the samples were plated with gold and observed with SEM.

For platelet activation test, the cell-seeded and cell-free electrospun grafts were treated with 400 μg of platelet-rich plasma (PRP) for 1 h at 37 °C. Briefly, rabbit whole blood was mixed with 3.8% anti-coagulant acid-citrated (9:1) and centrifuged at 1500 rpm for 15 min to prepare PRP. After that, all samples were centrifuged at 3500 rpm for 10 min to obtain the platelet poor plasma (PPP). The content of soluble P-selection (sP-selection) in the plasma was determined using rabbit P-Selection ELISA kit (FineTest, Wuhan, China) according to the protocol provided by the manufacturer.

### Live/dead stain assay

After 7 days of culture, the electrospun vascular constructs were stained with the live/dead assay kit. Briefly, the electrospun vascular constructs were washed with 1$\times$ PBS, and then, incubated for 30 min in a staining solution containing 0.4 μL Calcein-AM and 0.2 μL Ethidium homodimer-1 (EthD-1) [35]. After staining, the electrospun vascular constructs were washed with 1$\times$ PBS for three times, fixed with 4% paraformaldehyde at room temperature for 10 min, and the fixed solution was removed. The fixed electrospun vascular constructs were then dried in a freeze-drying oven (PE-1E-80, Shanghai Jipu Electronic Technology Co., Ltd) for 48 h. Finally, the electrospun vascular constructs were frozen embedded in a cryostat (Leica cm1950) and sliced (20 μm thickness). Confocal laser scanning microscope (CLSM, SP5, Leica, Germany) was used to observe the stained cells. The excitation/emission filter was set at 495/510–530 nm to observe the living cells (stained green), and the dead cells (stained red) were detected at 530/610–620 nm.

### Cell proliferation and viability assay

MTT assay was used to quantify the cellular metabolic activity in the tubular HA/collagen nanofibrous scaffold. To evaluate the cell proliferation of vascular ECs and SMCs respectively, tubular HA/collagen nanofibrous scaffolds of 0.5 cm in length were culturing with vascular ECs alone, and SMCs alone in tubular HA/collagen nanofibrous scaffold separately. After seeding, they were transferred into 96-well plates and cultured in 37 °C in an incubator with a humidified 5% CO_2_ atmosphere. At days 1, 3, 5 and 7, the original culture medium in 96-well plate was sucked out and replaced with 100 μL fresh whole cell culture medium containing 20 μL MTT reagent. After incubation for 1 h, the scaffolds were removed from 96-well plates, and the absorption values were measured. The medium was measured at 490 nm with full-wavelength micro-plate reader (Multiskan GO, Thermo Fisher, Waltham, USA). Cells cultured on standard plate were used as positive control. All the samples are in quadruplicate.

As described in the above step, CCK-8 kit was used to analyze the cell viability of vascular ECs and SMCs respectively, in tubular HA/collagen nanofibrous scaffold. Cells cultured on standard plate were used as positive control. Briefly, at the end of the culturing, the medium was removed and washed once with 1× PBS, then each sample was incubated with 100 μL DMEM contains 10 μL CCK-8 for 3 h to form water dissoluble formazan. Then, 100 μL of this formazan solution were collected from each sample and added to one well of a 96-well plate. The absorbance at 450 nm (calibrated wave) was determined using a full-wavelength micro-plate reader. All the samples are in quadruplicate.

### Surgical implantation and graft patency test

All animals were performed according to the guidelines approved by the Institutional Animal Care and Use Committee of Guangzhou Medical University. In vivo experiments were performed on 6 New Zealand male rabbits (2.8 kg, 12 weeks old). Rabbits were treated with aspirin for one week during and after implantation. Under general anesthesia, the left carotid artery was exposed through a middle incision in the neck. Heparin (100 units/kg) was injected intravenously before partial occlusion of the vessel with the side bite forceps. A 4 cm long portion of the left carotid artery was replaced with an electrospun vascular graft, and then the graft was sutured end-to-end to the left carotid artery.

Following implantation, bioengineered grafts were examined every 2 weeks for patency using a Sequoia ultrasound device (Siemens Inc., ACUSON Sequoia, Germany) for double ultrasound system, and the diameter and patency of the middle section of the grafts were recorded.

### Histological assessment

Six weeks after implantation, the animals were scheduled euthanasia by CO_2_ inhalation, and the explants were washed heparinized saline, and then fixed with 4% paraformaldehyde for 24 h, dehydrated with ethanol gradient solutions, and embedded in paraffin. The cross-section of the explants was cut into 6 μm sections, as published methods; the serial sections of the same cross-section were stained with H&E and EVG stain kit, respectively, using standard methods [29, 30, 35]. The structural characteristics, cell infiltration and tissue remodeling were examined. Parallel specimens were analyzed for immunofluorescence (IF) assessments utilizing primary antibodies to select antigens including CD31 [mouse immunoglobulin G (IgG), 1:100 dilution] and α-SMA (rabbit IgG, 1:100 dilution). In short, following de-paraffinization and anti-gen heat retrieval, the explant sections were permeabilized with 0.15% Triton X-100-PBS for 15 min and blocked in 1$\times$ PBS solution contains 1% BSA for 1 h at room temperature. The sections were treated with primary antibodies at specified dilutions overnight at 4 °C. After twice washes in 1$\times$ PBS at RT, sections were then incubated for 1 h at RT with species-matched Cy3 or Alexa Fluor-488-conjugated secondary antibodies. Following specimen washing with PBS, nuclei were counterstained with DAPI. Sample visualization was performed with CLSM and representative fields were acquired with LAS AF lite software. Histomorphometric evaluations (*n* = 3 animals/group) were performed on six independent microscopic fields using published methods to quantify the percentage of elastic fiber area, and the CD31 positive as well as α-SMA positive area in experimental cohorts [30, 35].

### Statistical analyses

Statistical analysis was performed using GraphPad Prism 5.0 (GraphPad Software, La Jolla). The results of all quantitative analysis are expressed as mean $\pm$ standard deviation (s.d.). Newman–Keuls post-hoc tests were employed to compare the difference between the research groups. Obtained *p*-values (*p < 0.05, **p < 0.01, ***p < 0.001) were considered to be statistically significant.

## Results

### Characterization of the tubular HA/collagen nanofibrous scaffold

**Fig. 1:**
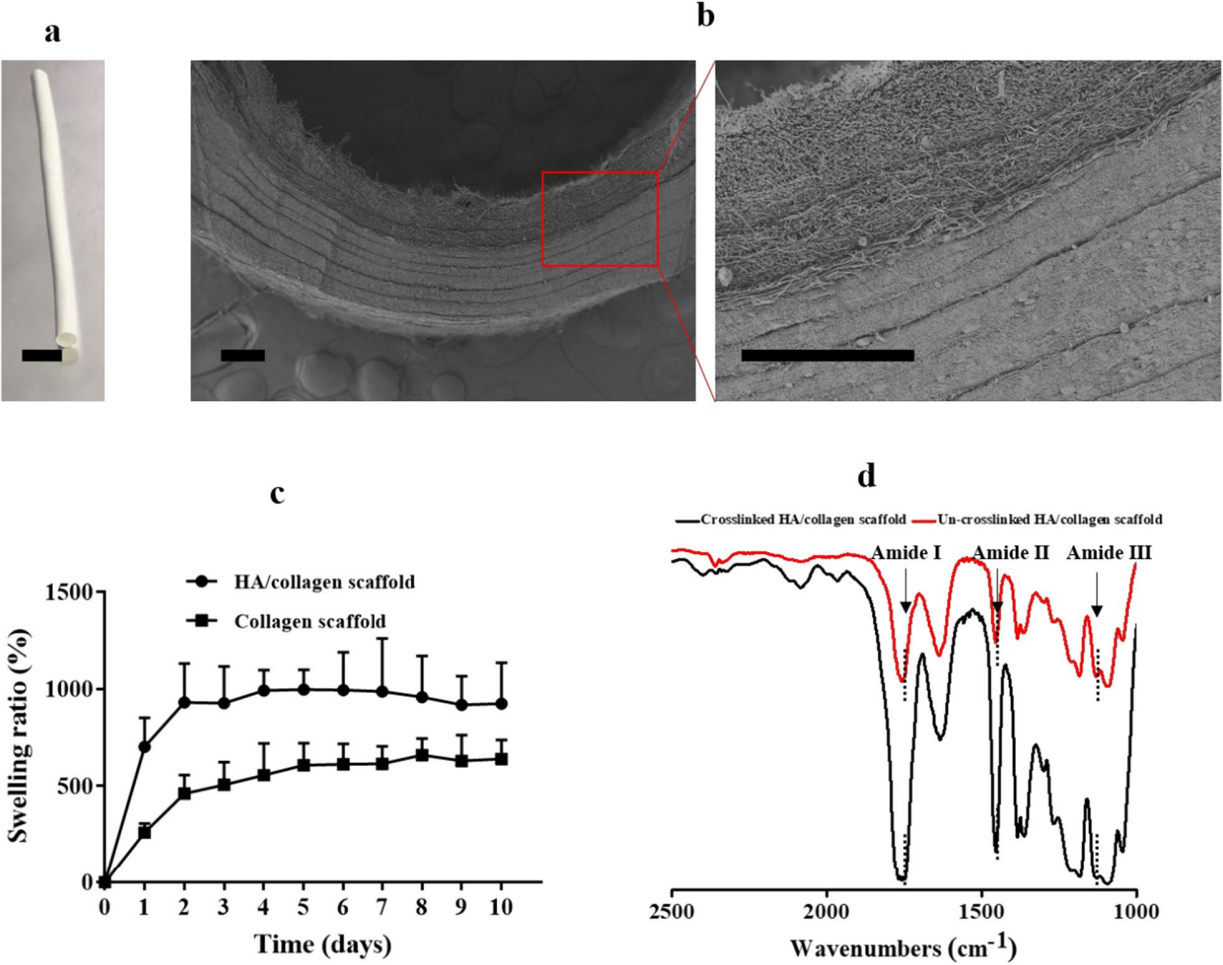
Biophysical characteristics of the tubular HA/collagen nanofibrous scaffold. **a** The frontal view and **b** SEM micrograph of the cross-section of a tubular HA/collagen nanofibrous scaffold. Scale bars, **a** 1 cm, **b** 200 μm. **c** The swelling ratios of a tubular HA/collagen and collagen nanofibrous scaffolds in pH 7.4 at 37 °C. **d** ATR-FTIR of a tubular HA/collagen before and after cross-linking

We fabricated tubular HA/collagen composite nanofibrous scaffolds by sequential electrospinning (Fig. 1a). The composite scaffold is a white hollow tube with a length of 70 mm, an inner diameter of 3 mm (Fig. 1a). The wall thickness of the obtained tubular scaffolds is in the range of (0.65–1) mm (Additional file 1: Table S1). The cross-section (Fig. 1b) consists of two main layers. The nanofibers on the inner wall are loose and the nanofibers on the outer wall are tightly layered. The average diameters measured for the HA/collagen fibers are in the range of (905 $\pm$ 113) nm (Additional file 1: Table S1). To match the sizes of native blood vessels, they can be cut into different lengths. Hollow nanofiber scaffolds with different inner diameters can be obtained by changing the outer diameter of stainless steel in the receiving device. To produce wall thickness similar to those of human arteries, the thickness of inner wall layer and outer wall layer can also be adjusted by feed time (Additional file 1: Table S1).

The porosity of tubular composite scaffolds was 82.7%, and the average pore diameter of inner wall (Fig. 1b) and outer wall were (2.139 $\pm$ 81) μm and (2.159 $\pm$ 77) μm, respectively, indicating that tubular fiber scaffolds had a microstructure conducive to effective nutrient diffusion. The swelling ratio of tubular HA/collagen nanofibrous scaffolds reached equilibrium after 48 h of immersion. The volume increased by 8.6 times and remained unchanged in the whole experiment (10 days) (Fig. 1c), indicating that the water absorption property was rapidly stable.

ATR-FTIR analysis was used to examine the secondary structure changes induced by glutaraldehyde vapor crosslinking (Fig. 1d). Repeated units of peptides and proteins usually produce three characteristic infrared absorption bands: amide I (1600–1690 cm^−1^), amide II (1480–1575 cm^−1^) and amide III (1229–1301 cm^−1^) [9, 20]. The characteristic absorption band of α-helix (1655 cm^−1^), random coil (1645 cm^−1^), and β-folding (1630 cm^−1^) is located in amide I, which represents the C=O stretching vibration of amide group [17]. The peak value of un-crosslinked tubular HA/collagen nanofibrous scaffold in amide I absorption was 1645 cm^−1^, and the displacement of glutaraldehyde vapor crosslinked HA/collagen nanofibrous scaffold was 1630 cm^−1^. This change indicates that glutaraldehyde vapor crosslinking may result from random coil to β-folding transition (Fig. 1d).

The tensile strength of tubular HA/collagen nanofibrous scaffolds were 1.37 $\pm$ 0.7 MPa, which was close to that of native artery (~ 1.4 MPa). Based on the above observations, tubular HA/collagen nanofibrous scaffold have good biophysical properties, such as stiffness, structure and topography matching with natural arteries.

### Cell adhesion in scaffold and anti-platelet adhesion

**Fig. 2:**
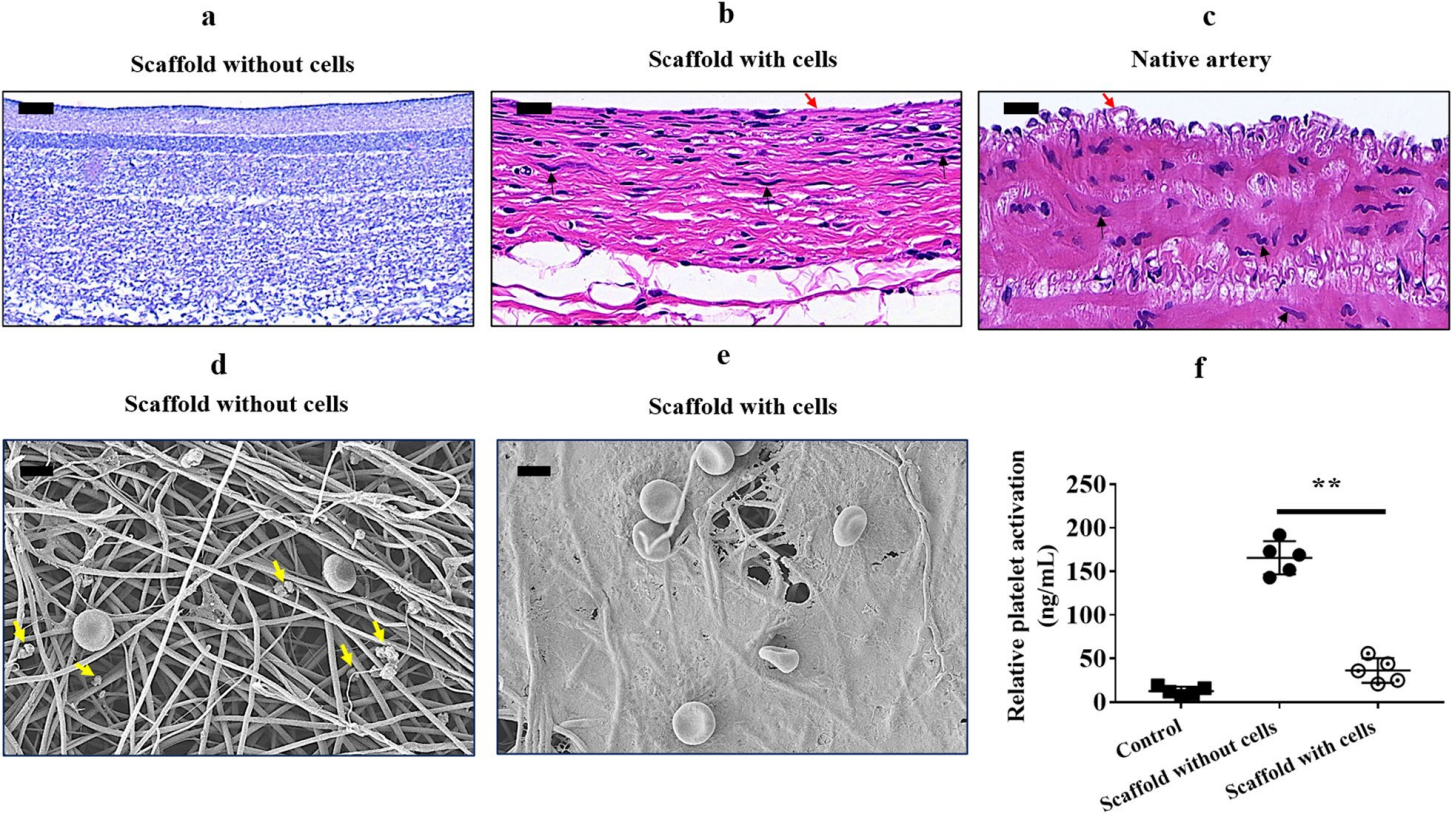
Cell adherence and resistance to platelet adherence assessments. H&E staining of the cross-section of a tubular HA/collagen nanofibrous scaffold before (**a**) and after (**b**) cell seeding, and a rabbit native carotid artery (**c**). Vascular ECs and SMCs adhere to the lumen and exterior, respectively, of the tubular HA/collagen nanofibrous scaffold after 7 days cell seeding. A thin layer of flat ECs (red arrows) lining the lumen, bundles of filaments with dense bodies (black arrows) are characteristic of SMCs. Scale bars, 20 μm. SEM micrographs of HA/collagen scaffold without (**d**) and lined with vascular ECs (**e**) after exposure to blood for 15 min. Yellow arrows show the morphology of rabbit platelets. Scale bars, 4 μm. **f** Quantification of platelet activation by measuring the expression of sP-selectin release in rabbit PRP following incubation with HA/collagen scaffold without and lined with vascular ECs (*n* = 4). **p $<$ 0.01

To test the ability of tubular HA/collagen composite nanofibrous scaffolds to support the growth of vascular ECs and SMCs, we first seeded rabbit vascular ECs on the inner surface of the scaffolds, and then, SMCs on the outer surface of the scaffolds. Compared to scaffolds that had not been seeded with cells (Fig. 2a), ECs can adhere to the surface of the inner wall of the scaffold and form a fusion layer in the inner cavity of the scaffold (Fig. 2b), while smooth muscle cells present a multilayer structure outside the scaffold, similarly to the structure of native blood vessel (Fig. 2c). This result indicates that the porosity of luminal endothelialized scaffolds facilitate the infiltration of SMCs.

To evaluate whether ECs on electrospun scaffolds can resist platelet adhesion, bare scaffolds and endothelialized scaffolds were exposed to arterial flow for 15 min. SEM micrography showed that bare scaffolds without ECs showed a large number of platelet adhesion (Fig. 2d). Interestingly, SEM evidenced a fusion layer of ECs on scaffolds able to resist adhesion of platelets (Fig. 2e). This observation was further confirmed by quantification of platelet activation assay (Fig. 2f). These findings indicate that tubular HA/collagen electrospun scaffolds can support the growth of cell types from native blood vessels and vascular ECs that show proper function in resisting platelet adhesion.

### Cell proliferation and viability in scaffold

**Fig. 3:**
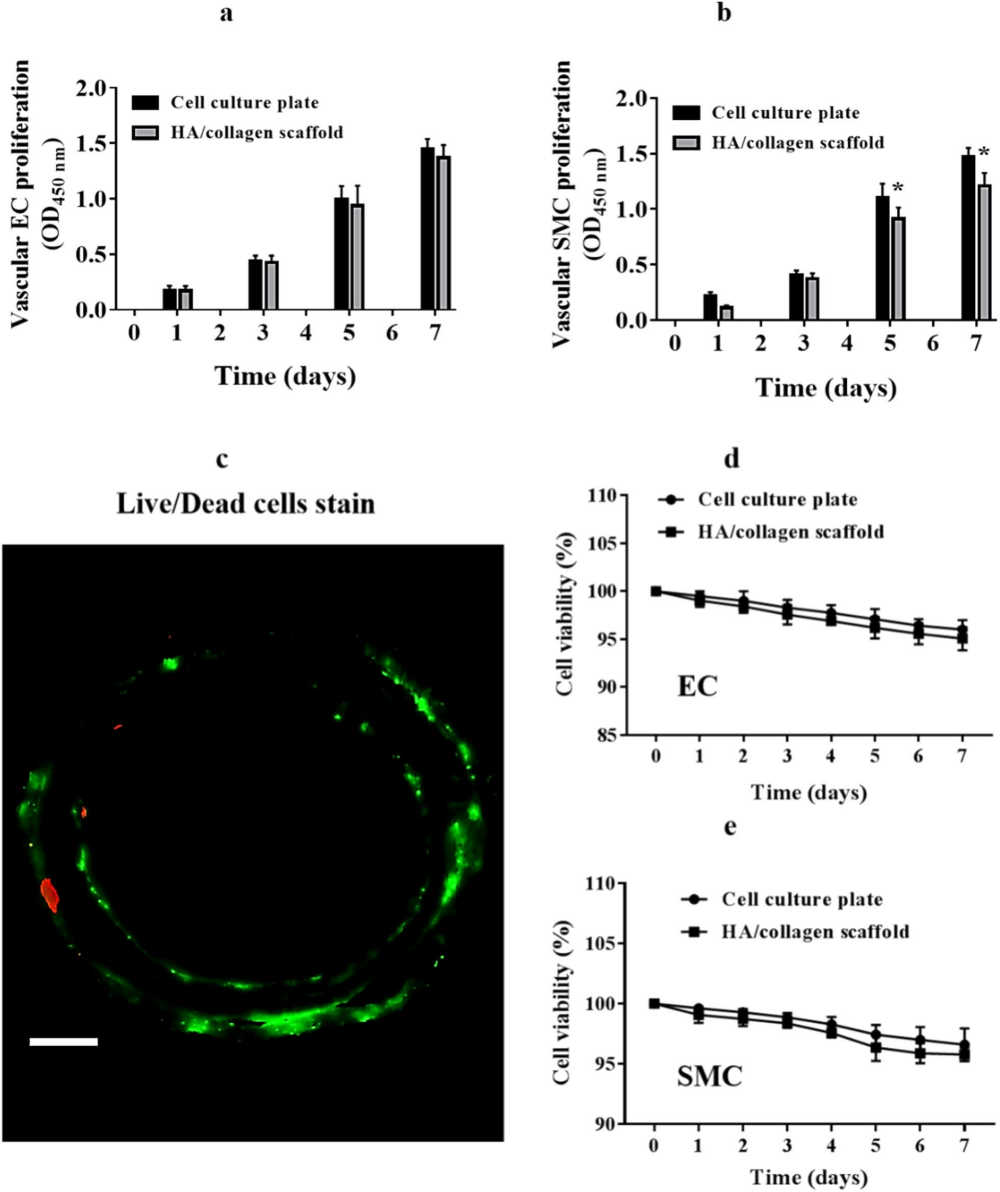
The proliferation and viability of vascular ECs and SMCs in the tubular HA/collagen nanofibrous scaffolds. The proliferation of vascular EC in the inner wall (**a**) and SMC in the outer (**b**) wall surface of a tubular HA/collagen nanofibrous scaffold and a cell culture plate (positive control) during the period of 7 days (*n* = 5). *p $<$ 0.05. Live/dead stain of ECs and SMCs (**c**) in a tubular HA/collagen nanofibrous scaffold on day 7 after cell culturing. Scale bars, 500 μm. Quantification of live ECs (**d**) and SMCs (**e**) over a period of 7 days (*n* = 4)

To determine whether tubular HA/collagen nanofibrous scaffolds affect the cellular behavior of vascular ECs and SMCs; we used MTT to analyze the growth of ECs or SMCs on the scaffolds. Vascular ECs (Fig. 3a) and SMCs (Fig. 3b) could proliferate on the inner and outer surface of tubular scaffolds for more than 7 days, indicating that vascular ECs and SMCs were proliferating well in the HA/collagen composite scaffolds. The Live/dead cell staining micrograph further confirmed these results (Fig. 3c). Moreover, the cytotoxicity of tubular HA/collagen nanofibrous scaffolds was determined by CCK-8 assay. When vascular ECs and SMCs were grown on tubular HA/collagen nanofiber scaffolds for 7 days in vitro, respectively, cellular viability was more than 95% (Fig. 3d, e). These results indicate that tubular HA/collagen nanofibrous scaffolds can provide an ideal microenvironment for the proliferation of both vascular ECs and SMCs.

### Structural stability of celluarized tubular HA/collagen nanofibrous grafts in vivo

**Fig. 4:**
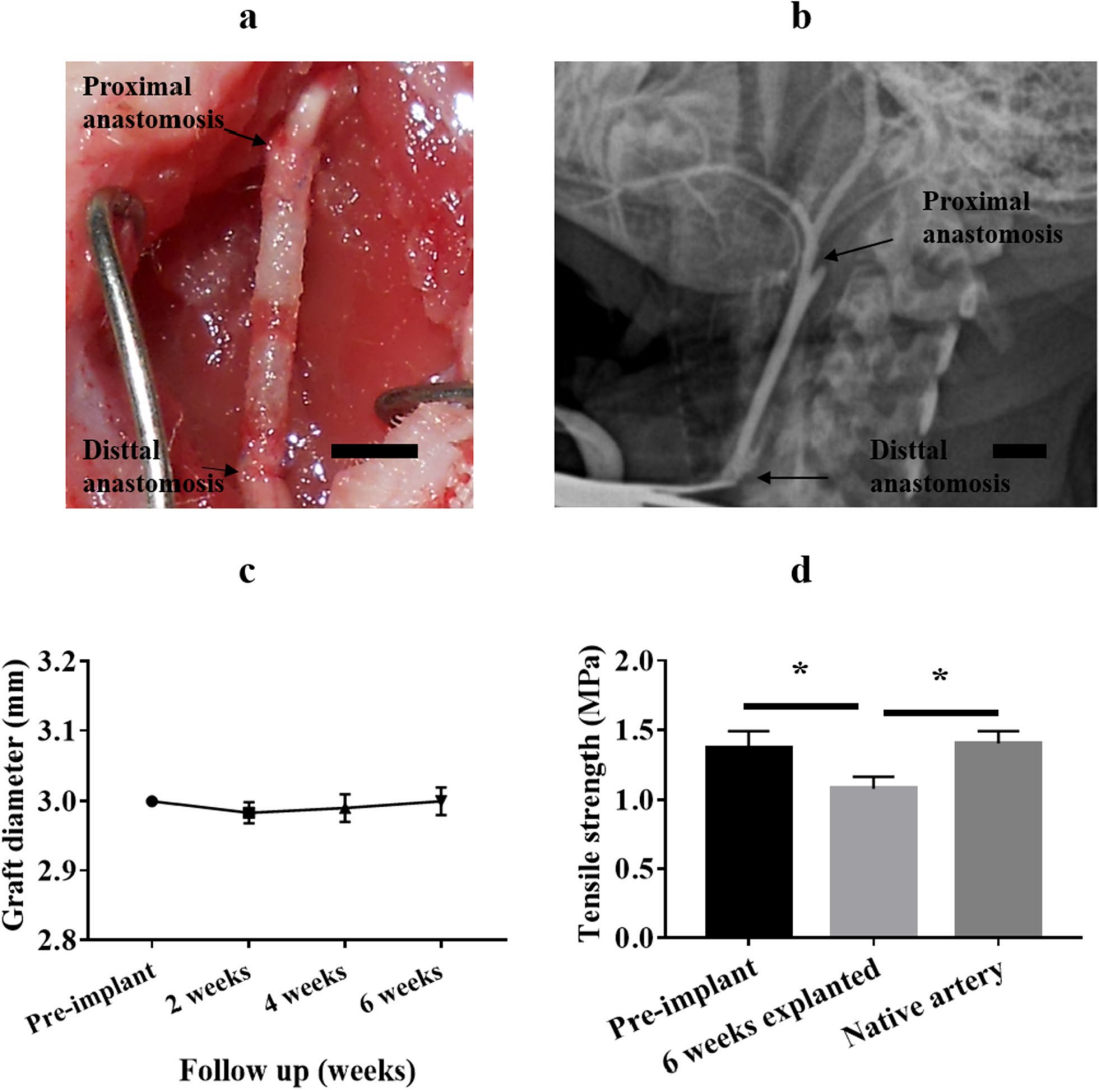
Surgical implantation and outcome of cellularized tubular HA/collagen nanofibrous scaffold graft. An end-to-end anastomosis procedure with the nanofibrous scaffold graft sutured to the carotid artery in rabbit (**a**). A representative angiography image shows absence of aneurysm along the entire length of the graft (**b**). A summary of ultrasound measurements revealed a stable lumen caliber for the duration of the 6 weeks study. Black arrows show margins of tissue-engineered blood vessel. Scale bars, **a**, **b** 1 cm, **c** tensile strength was compared between pre-implant nanofibrous scaffold strips, 6 weeks retrieved explant strips and carotid artery strips (*n* = 3). *p $<$ 0.05

The baseline structural integrity and function of scaffold grafts were evaluated in a rabbit carotid artery replacement model, in order to determine the applicability of tubular HA/collagen nanofibrous vascular scaffolds. At the time of implantation, the graft showed no sign of bleeding leakage (Fig. 4a). No bleeding or neurological complications occurred throughout the research. To determine whether the implanted cellularized tubular HA/collagen nanofibrous grafts can maintain structural integrity and resist the development of aneurysms, bifunctional ultrasound was performed every 2 weeks to evaluate the blood flow patency (Fig. 4b) and graft diameter (Fig. 4c). Throughout the study, the graft maintained a constant lumen diameter as measured by ultrasound (Fig. 4c). These results indicate that cellularized tubular HA/collagen nanofibrous grafts can maintain structural integrity under normal hemodynamic conditions.

To supplement the structural integrity find by surgery and ultrasound, we quantitatively evaluated the biomechanical strength of the graft at the end of the 6-week in vivo arterial replacement experiment. After retrieval, strip-shaped membranes with a length of 40 mm and a width of 9.5 mm (circumferential length of the tubular scaffolds) were obtained by transverse cutting along the wall of the graft. The results were compared with those of the scaffold before implantation and natural artery. As shown in Fig. 4d, although there was a slight decrease in the tensile strength of the grafts during the 6-week study period, they were still close to the natural arteries. This indicates that the cellularized tubular HA/collagen nanofibrous grafts can maintain a considerable degree of tensile strength even when exposed to in vivo conditions.

### Vascular reconstruction assessment

**Fig. 5:**
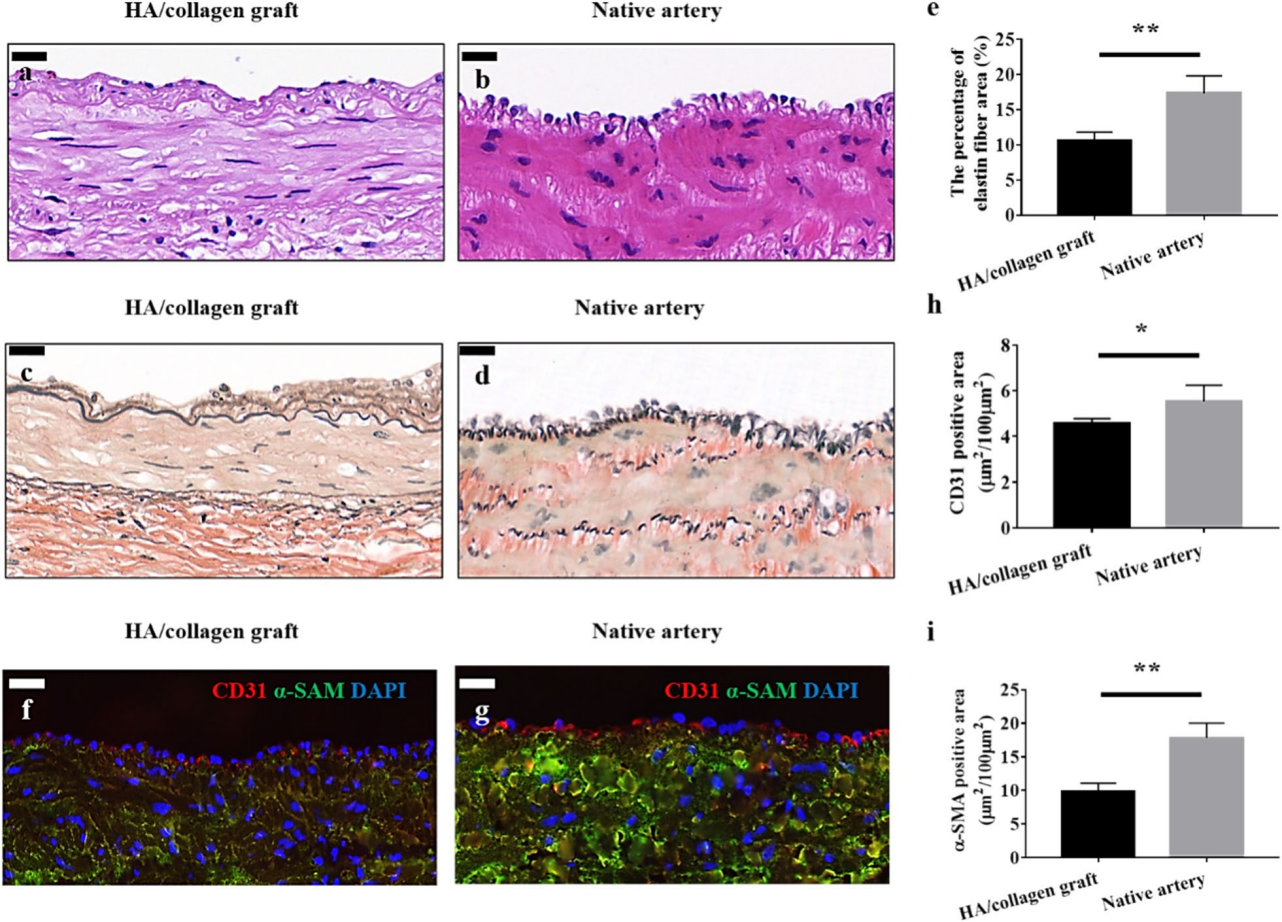
Vascular grafts fabricated with cellularized tubular HA/collagen nanofibrous scaffold facilitates vascular reconstruction. H&E staining (**a**), VVG staining (**c**) and fluorescent staining (**f**) the cross-section of a retrieved tubular HA/collagen nanofibrous grafts 6 weeks after transplant at 10$\times$ (**a**, **c**, **f**) compared with rabbit carotid artery (**b**, **d**, **g**). Scale bars, 20 μm. Fluorescence relative to vascular ECs (red), SMCs (green) and nuclei (blue). Quantification of the percentage of elastin area (**e**), CD31- (**h**) and α-SMA-positive (**i**) in the retrieved explants 6 weeks after transplant and carotid artery (*n* = 3). *p $<$ 0.05, **p $<$ 0.01

Histologically, H&E staining micrographs showed that the vascular grafts maintained the original morphology before and after implantation (Figs. 2b and 5a). Moreover, 6 weeks post-implantation, the morphology of ECs, lining the inner wall of the graft, is wavy and similar to that of the native artery (Fig. 5b). A small number of cells infiltrated into the outer wall of the graft (Additional file 1: Fig. S1). VVG staining showed that elastin fibers (the main component of vascular ECM) are formed along the lumen and smooth muscle on the wall (Fig. 5c) and comparable with the native tissue morphology of artery (Fig. 5d). Quantitatively, the percentage of elastin fibers in the graft was still slightly lower than that of naive arteries (Fig. 5e). This result is consistent with previous reports that the formation of elastic fibers in tissue-engineered vascular scaffolds is crucial to the success of tissue-engineered arteries [14, 35].

Next, parallel specimens of the retrieved grafts were analyzed by double-color IF staining (Fig. 5f). Compared with native artery (Fig. 5g), the lumen of vascular grafts made of cellularized tubular HA/collagen nanofibrous scaffold lined with a layer of CD31 positive cells (vascular ECs), α-SMA positive cells (vascular SMCs) were circumferentially distributed in the HA/collagen nanofibrous graft wall (Fig. 5f). Quantitatively, the content of CD31 positive cells and α-SMA was slightly lower than that of natural arteries, respectively (Fig. 5h, i). It is suggested that the vascular grafts made of cellularized tubular HA/collagen nanofibrous scaffolds can provide a full framework of 3D matrix in order to retain cells’ phenotypic shape and maintain their natural behavior and functionality.

## Discussion

The ultimate goal of tissue-engineered vascular scaffolds is to promote vascular reconstruction and functional recovery of damaged vessels. The effect of reconstruction is closely related to the biophysical and biochemical signals from scaffolds [20, 36–38]. In this study, collagen and HA were used as raw materials and a porous, hierarchical composite tubular vascular nanofibrous scaffold was successfully prepared by using the electrospinning technique. Among the characteristics of this composite scaffold, the peculiar mechanical properties of collagen in synergy with biological activity of HA can be fully harnessed. In vitro cell experiments confirmed that HA nanofibers on the inner surface of scaffolds can promote the adhesion of vascular ECs, while the porosity of scaffolds can promote the histomorphogenesis of vascular smooth muscle by promoting their infiltration and diffusion from outer wall surface. These results are consistent with previous reports highlighting that cell scaffold biophysical properties such as stiffness, structure and topography are critical for normal cell function [12, 13, 26, 37, 38]. Furthermore, the composite scaffold can provide a complete 3D nanofibrous matrix microenvironment for the proliferation of vascular EC and SMC. The scaffold was seeded with cells to obtain a cell filled tissue matrix before implantation. The advantage of this method is to obtain a biological relevant in vitro construct mimicking the native tissue functions.

One of the main reasons for the failure of small-diameter vascular transplantation is early embolism (thrombosis) [38–40]. The cause can be found in the luminal ECs loosing phenotype in 3D ECM environment after implantation, in turn decreasing ECM production and function [41, 42]. To match rabbit carotid artery size, we prepared a graft with an inner diameter of 3 mm. By changing the diameter of stainless steel rod in the electrospinning receiver, we can easily obtain tubular electrospun nanofiber scaffolds with different sizes. During the 6-week study period, all grafts made of cellularized tubular HA/collagen nanofibrous scaffolds remained patent, and ultrasound evaluation showed that the lumen diameter remained unchanged. These results indicate that the tubular HA/collagen nanofibrous grafts constructed in vitro can reproduce the antithrombotic function of native vascular endothelium. Further histology and IF staining confirmed that tubular HA/collagen nanofibrous grafts can support the attachment and growth of vascular EC and SMC under physiological conditions as well as maintain their phenotypic shape [42].

In the reconstructed blood vessels bridged by cellularized tubular HA/collagen nanofibrous scaffolds, new tissue was remodeled owing to the newly formed ECM and additional recruitment of endogenous cells. Compared with situation before implants, biomechanical measurements of the grafts after 6 weeks implantation showed a slight decrease in tensile strength, but still close to that of the natural aorta. These findings suggest that cellularized tubular HA/collagen electrospun grafts maintain structural integrity in vivo under normal hemodynamic conditions, at least in the second stage of tissue regeneration. It is worth noticing that the morphology and structure of the biomimetic vascular graft constructed on the cellularized tubular HA/collagen nanofibrous scaffold are close to the native artery under physiological conditions. However, the quantitative improvement in morphology and structure remains to be determined. At this stage, the biophysical integrity of the newly formed ECM was improved through tissue reorganization, degradation and re-synthesis [6, 42–48]. We expect that third stage regeneration can be achieved within 2 years.

## Conclusion

The tubular HA/collagen nanofibrous scaffolds prepared by sequential electrospinning showed good biocompatibility, high porosity and mechanical properties matching with native arteries. The biophysical and biochemical clues of tubular HA/collagen nanofibrous scaffolds provided three-dimensional microenvironment for the growth of vascular ECs and SMCs under physiological conditions, and maintain vascular patency highlighting regeneration and reconstruction in the in vivo model of rabbit. Future studies will examine the morphogenesis of these cellular tubular HA/collagen electrospun grafts during the tissue integration phase.

## Supplementary Information

**Additional file 1: Figure S1.** Vascular grafts fabricated with cellularized tubular HA/collagen nanofibrous scaffold facilitates vascular reconstruction. H&E staining the cross-section of a retrieved tubular HA/collagen nanofibrous grafts 6 weeks after transplant at 2.5$\times$ (upper panel) and 40$\times$ (lower panel) compared with rabbit carotid artery (b, d, g). Scale bars, 100 μm. **Table S1. **The size of the obtained electrospun tubular HA/collagen nanofibrous scaffolds.

## Data Availability

All data supporting this study are included in this article.
