# Supplementary material for: In vivo performance of electrospun tubular hyaluronic acid/collagen nanofibrous scaffolds for vascular reconstruction in the rabbit model
Source: J Nanobiotechnology. 2021 Oct 30;19:349. doi: 10.1186/s12951-021-01091-0 (PMC8557601; doi:10.1186/s12951-021-01091-0)
Supplement: Supplementary file 1 — Additional file 1: Figure S1. Vascular grafts fabricated with cellularized tubular HA/collagen nanofibrous scaffold facilitates vascular reconstruction. H&E staining the cross-section of a retrieved tubular HA/collagen nanofibrous grafts 6 weeks after transplant at 2.5$\times$× (upper panel) and 40$\times$× (lower panel) compared with rabbit carotid artery (b, d, g). Scale bars, 100 μm. Table S1. The size of the obtained electrospun tubular HA/collagen nanofibrous scaffolds. [file 12951_2021_1091_MOESM1_ESM.docx]

## Additional file 1

*In vivo* performance of electrospun tubular hyaluronic acid/collagen nanofibrous scaffolds for vascular reconstruction in the rabbit model

Yuqing Niu^a^, Massimiliano Galluzzi^b^, Ming Fu^a^, Jinhua Hu^a^, Huimin Xia^a,*^

^a^ Department of Pediatric Surgery, Guangdong Provincial Key Laboratory of Research in Structural Birth Defect Disease, Guangzhou Women and Children's Medical Center, Guangzhou Medical University, Guangzhou 510623, Guangdong, P.R. China.

^b^ Materials Interfaces Center, Shenzhen Institutes of Advanced Technology, Chinese Academy of Sciences, Shenzhen 518055, Guangdong, P.R. China.

*Correspondence: [xia-huimin@foxmail.com](mailto:xia-huimin@foxmail.com)


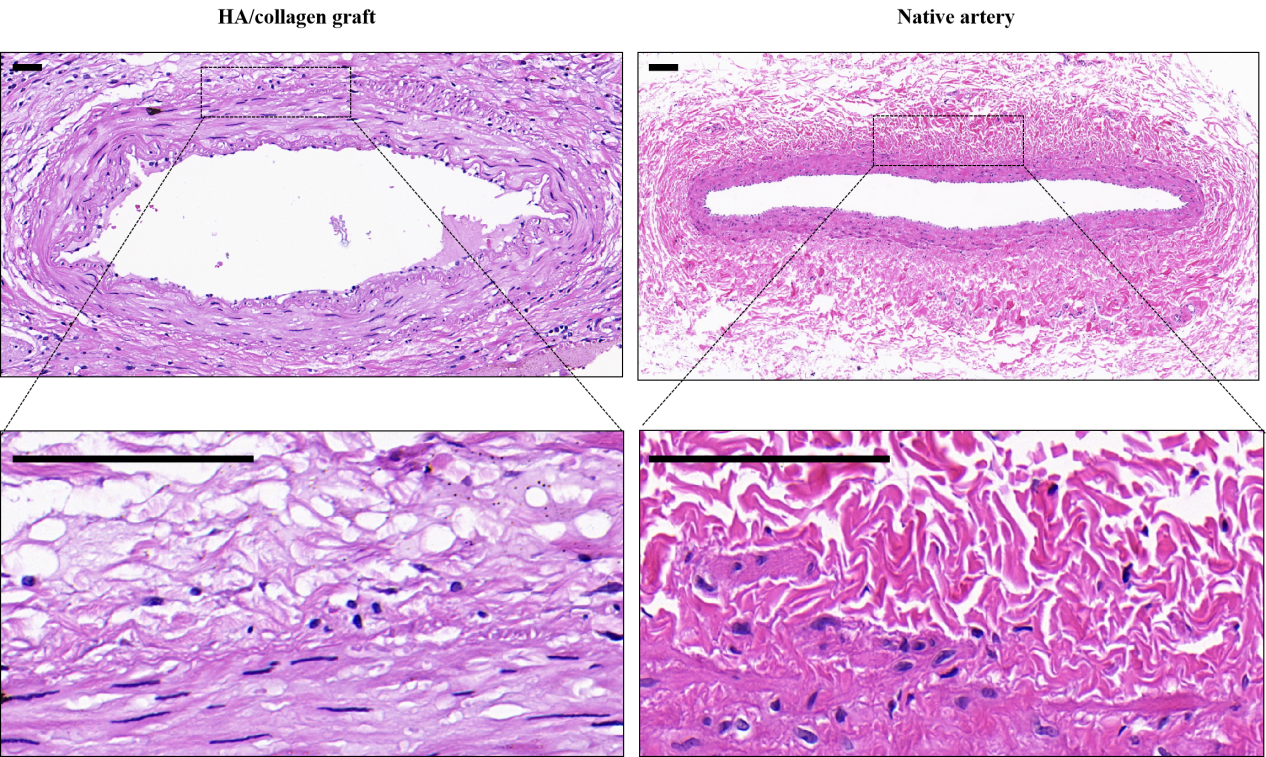


***Fig. S1.*** *Vascular grafts fabricated with cellularized tubular HA/collagen nanofibrous scaffold facilitates vascular reconstruction. H&E staining the cross-section of a retrieved tubular HA/collagen nanofibrous grafts 6 weeks after transplant at 2.5*$\times$*(upper panel) and 40*$\times$ *(lower panel) compared with rabbit carotid artery (b, d, g). Scale bars, 100 μm.*

***Table S1.*** *The size of the obtained electrospun tubular HA/collagen nanofibrous scaffolds.*

| ***Sample*** | ***Length***  ***(cm)*** | ***Internal diameter***  ***(mm)*** | ***Wall thickness***  ***(mm)*** | ***Fiber diameter***  ***(nm)*** |
| --- | --- | --- | --- | --- |
| *HA/collagen* | *(0.5-9) cm* | *(2.6-3.0) mm* | *(0.6-1.0) mm* | *(905*$\pm$*113) nm* |
